# The impact of garlic and its active metabolites on degenerative musculoskeletal diseases

**DOI:** 10.3389/fphar.2026.1847433

**Published:** 2026-06-10

**Authors:** Nana Cheng, Jieyou Zhao, Chenhao Feng, Zhaoyang Li, Yunjia Song, Xutao Sun

**Affiliations:** School of Basic Medical Sciences, Heilongjiang University of Chinese Medicine, Harbin, China

**Keywords:** garlic, inflammation, intervertebral disc degeneration, osteoarthritis, osteoporosis, oxidative stress, sarcopenia

## Abstract

With the accelerating global population aging, the incidence of degenerative musculoskeletal diseases (such as osteoarthritis, osteoporosis, intervertebral disc degeneration and sarcopenia) continues to rise, posing a significant public health challenge. Current conventional therapeutic approaches, while alleviating symptoms, are often accompanied by side effects and struggle to reverse the pathological process. Garlic and its various active metabolites (such as allicin, S-allylmercaptocysteine, diallyl sulfide and diallyl disulfide, etc.) have been confirmed to possess multiple biological activities, including anti-inflammatory, antioxidant effects, regulation of signaling pathways, and maintenance of extracellular matrix homeostasis. Numerous studies have demonstrated that the active metabolites of garlic can intervene in degenerative musculoskeletal diseases by regulating multiple signaling pathways such as PI3K/Akt/NF-κB, RANKL/RANK/OPG, Wnt/β-catenin, and Akt/mTOR, significantly delaying the progression of the diseases. Therefore, this review summarizes the regulatory effects and potential mechanisms of garlic and its bioactive metabolites on degenerative musculoskeletal diseases, aiming to provide a scientific basis for the further development of adjunctive therapeutic strategies based on garlic active metabolites.

## 1 Introduction

Degenerative musculoskeletal diseases refer to a category of diseases characterized by chronic progressive degeneration or deterioration of the bones, cartilage, joints, and surrounding tissues (Wen et al., 2023). These conditions primarily encompass osteoarthritis, osteoporosis, intervertebral disc degeneration, and sarcopenia (Wen et al., 2023). Their clinical manifestations are predominantly characterized by progressively worsening pain, restricted mobility, and functional decline, accompanied by structural degenerative changes such as cartilage deterioration, reduced bone mineral density, and loss of muscle mass. In severe instances, these conditions can cause disabilities, greatly impacting patients’ quality of life and physical health. These diseases predominantly affect middle-aged and elderly populations and are a major factor contributing to functional impairment and reduced quality of life in older adults. The global aging process has led to a yearly rise in these disorders, increasing healthcare resource use and imposing significant socioeconomic burdens, thus representing a major global public health issue (Li and Chen, 2019). Current conventional treatment methods can alleviate symptoms to a certain extent, but they demonstrate limited efficacy in reversing pathological progression and are associated with issues such as drug dependence, disease recurrence, and surgical risks. Recent studies have indicated that natural metabolites found in onions, garlic, and tomatoes possess potential therapeutic effects against osteoarthritis (Salem et al., 2023), (Yang J. et al., 2020), with fewer side effects and a higher safety profile compared to traditional drugs. Consequently, exploring and developing novel, safe, and effective treatment strategies based on natural products, and delving into their regulatory mechanisms, holds significant scientific and clinical importance.

**FIGURE 1:**
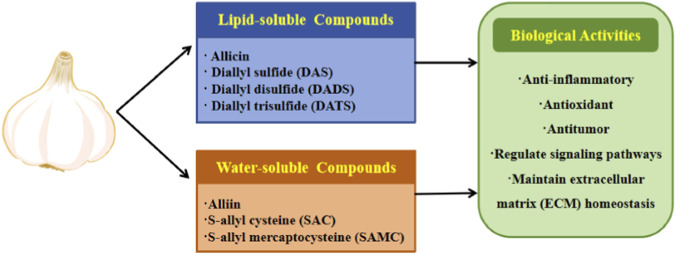
The classification and effects of active metabolites of garlic.

Garlic is a widely used aromatic herbaceous plant, valued both as a culinary ingredient and as a complementary treatment for various diseases (El-Saber Batiha et al., 2020). Garlic extracts and bioactive metabolites, rich in organosulfur compounds, polyphenols, flavanols, flavonoids, saponins, and tannins, exhibit therapeutic potential in orthopedic, cardiovascular, and metabolic diseases. They demonstrate multiple biological activities, such as analgesic, antithrombotic, antioxidant, anti-inflammatory, and antitumor effects (Tesfaye, 2021), (Huang et al., 2023), (Trio et al., 2014), (Pandey et al., 2023). Garlic’s main bioactive metabolites, organosulfur compounds, are divided into lipid-soluble types such as allicin, diallyl sulfide (DAS), diallyl disulfide (DADS), and diallyl trisulfide (DATS), and water-soluble types such as S-allyl cysteine (SAC) and S-allyl mercaptocysteine (SAMC) (Thomson and Ali, 2003) (Figure 1). Among these, allicin is a key bioactive substance. Upon enzymatic hydrolysis by alliinase, alliin is converted to allicin, which subsequently serves as a precursor for the generation of numerous bioactive derivatives, including DAS, DATS, and DADS (Sarvizadeh et al., 2021). Garlic and its bioactive metabolites exhibit multifaceted regulatory potential in degenerative musculoskeletal diseases. DAS has been shown to safeguard damaged chondrocytes by suppressing nuclear factor kappa-B (NF-κB) activation (Lee et al., 2009). Allicin mitigates IL-1β-induced inflammation in chondrocytes, reduces cartilage matrix degradation, and preserves cartilage tissue, thus slowing osteoarthritis progression (Qian et al., 2018). Clinical surveys indicate that garlic extract could be an adjunctive therapy for osteoarthritis (Zochling et al., 2004), (Dehghani et al., 2018), (Salimzadeh et al., 2018), emphasizing its potential value in treating degenerative musculoskeletal diseases. Accordingly, this review summarizes the regulatory effects of garlic and its bioactive metabolites, including their anti-inflammatory, antioxidant, and multi-signaling pathway modulatory activities, aiming to offer innovative research directions for natural product-based therapeutic strategies against degenerative musculoskeletal diseases (Table 1).

**TABLE 1:** Garlic and garlic derivatives and degenerative musculoskeletal diseases. ↓: downregulation, ↑: upregulation.

Diseases	Garlic derivatives	Mechanisms	Publication date	References
Osteoarthritis (OA)	Garlic Extract	——	2004	Zochling et al. (2004)
	DAS	inhibit NF-κB pathway COX-2↓	2009	Lee et al. (2009)
	DADS	MMPs,MMP-1 MMP-13↓	2010	Williams et al. (2010)
	Allicin	the proportion of cells in G0/G1 phase↓ the proportion of cells in S phase cyclin D1,CDK4 CDK6↑	2015	Li et al. (2015)
	SAMC	inhibit NF-κB pathway; MMP-1,MMP-9 MMP-13 MMPs/TIMP-1 C2C,TNF-α↓ TIMP-1↑	2017	Yang et al. (2017)
	Allicin	inhibit PI3K/Akt/NF-κB pathway iNOS,COX-2,NO prostaglandin E2 MMP-13 ADAMTS-5↓ Col II↑	2018	Qian et al. (2018)
	garlic supplementation	Serum resistin concentration↓	2018	Dehghani et al. (2018)
	garlic supplementation	——	2018	Salimzadeh et al. (2018)
Osteoarthritis (OA)	DADS	inhibit NF-κB-NFATc1 pathway TNF-α,IL-1β,IL-6 NO↓	2019	Yang et al. (2019)
	Allicin	active Keap1/Nrf2 pathway; iNOS,NOX-4,IL-6,MMP-13,TNF-α Keap1↓ GPX3,GPX4,CAT,GST,Nrf2,GAGs SOX-9,aggrecan, Col II,Nrf2,p-Nrf2↑	2020	Yang et al. (2020a)
	SAMC	inhibit NOX4/NF-κB pathway; MMP-2 MMP-9,MMP-13 IL-1β,TNF-α,IL-6 C2C,CTX-II,COMP COX-2,iNOS,NOX4↓ Col II,TIMP-1↑	2020	Yang et al. (2020b)
	Allium sativum	MMP-13,NO,NF-κB p65,IL-6,TNF-α COX-2,iNOS↓ Col II↑	2023	Salem et al. (2023)
	Garlic-derived Exosomes	inhibit MAPK pathway MMP-3,MMP-9 TNF-α↓; aggrecan Col II↑	2024	Liu et al. (2025)
Osteoporosis (OP)	Garlic oil	total cholesterol level, serum alkaline phosphatase, serum tartrate resistant acid phosphatase↓	2004	Mukherjee et al. (2004)
	Garlic oil	urinary calcium, phosphate, creatinine and hydro-xyproline Ca:Cr ratio, serum cholesterol, serum alkaline phosphatase Serum tartrate-resistant acid phosphatase↓; bone calcium, phosphate↑	2006	Mukherjee et al. (2006b)
	Garlic oil	intestinal mucosal calcium transference intestinal mucosal alkaline phosphatase activity, intestinal mucosal calcium ATPase activity bone ash calcium,phosphate content, serum estradiol hormone↑	2006	Mukherjee et al. (2006a)
	Garlic oil	IL-6,TNF-α,nitrite↓ CAT,serum estradiol,SOD TRAP↑	2007	Mukherjee et al. (2007)
Osteoporosis (OP)	Garlic Tablet	TNF-α↓	2012	Mozaffari-Khosravi et al. (2012)
	Allicin	promote PI3K/AKT and CREB/ERK pathway cytochrome c caspase-3,caspase-9,ROS↓	2016	Ding et al. (2016)
	Alliin	inhibit c-Fos-NFATc1 pathway ROS,Nox1,TRAP OSCAR DC-STAMP OC-STAMP RANKL,CD9 MMP-9↓	2016	Chen et al. (2016)
	Garlic Tablet	PCO,MDA,AOPP↓ TAC↑	2017	Ahmadian et al. (2017)
	Allicin	CTX↑	2020	Liu et al. (2020)
	Garlic Rhizoma	TC, LDL-C,5-HIAA 5-HT↓ SOD, ERα,ERβ↑	2022	Xiong et al. (2022)
	DATS	sphingosine, sphinganine,Rankl, TRAF6↓ BV/TV,Tb Runx2,β-catenin↑	2023	Zhang et al. (2023)
Intervertebral Disc Degeneration (IVDD)	Aged Garlic Extract	MDA↓ SOD↑	2012	Cemil et al et al. (2012)
	Aged Garlic Extract	MDA,NO,TNF-α IL,caspase-3↓ SOD,GSH-Px,CAT↑	2016	Cemil et al. (2016)
	Allicin	inhibit p38-MAPK pathway AOPP,MDA, caspase-3↓ MTP↑	2020	Xi et al. (2020)
	Allicin	Bax,caspase-3, caspase-9,ROS, ADAMTS-5, MMP13↓ Bcl-2,col2a1, aggrecan↑	2025	Zhang et al. (2025)
Sarcopenia (SP)	Aged Black Garlic	active mTOR/Akt/p70S6K pathway body weight increase rates,LDL,TG,CHO,GOP,GPT,PPARγ C/EBP,Atrogin-1, MURF-1↓ UCP-1, MyHC,PGC1-α↑	2023	Damluji et al. (2023)
	Raw Garlic	——	2025	Wang et al. (2025)

## 2 Literature search strategy

This narrative review aimed to provide a comprehensive overview of the current understanding of “The Impact of Garlic and Its Active Metabolites on Degenerative Musculoskeletal Diseases” comprehensively. Relevant studies were identified by searching the PubMed database. Keywords applied in the retrieval process included “Garlic”, “Allicin”, “Osteoarthritis”, “Osteoporosis”, “Intervertebral Disc Degeneration”, and their related synonyms. The search was primarily focused on articles published between 2004 and 2025, an emphasis on original research articles and clinical reports in the field. The inclusion of studies was based on their relevance to the core themes of this review, prioritizing original research and authoritative consensus statements. Given the considerable heterogeneity and methodological diversity of the existing literature on this subject, conditions for quantitative synthesis are not yet met. Therefore, a narrative review approach was adopted to integrate theoretical developments and research findings from a more comprehensive perspective. As a result, this review did not follow the PRISMA guidelines or other formal systematic review protocols.

## 3 The impact of garlic and its active metabolites on osteoarthritis regulation

Osteoarthritis (OA) is a degenerative joint disease characterized by the progressive deterioration of articular cartilage. The primary clinical symptoms are pain, swelling, and joint stiffness, significantly hindering patient mobility. The pathological characteristics of OA encompass degradation of articular cartilage, changes in subchondral bone, and inflammation of the synovium (Samuels et al., 2008). OA development is influenced by various risk factors such as joint injury history, overuse, aging, and being overweight, with a higher incidence observed in women compared to men. The pathological mechanism of OA focuses on degenerative changes in articular cartilage, involving interconnected processes like inflammatory cytokine activation, extracellular matrix (ECM) metabolic imbalance in chondrocytes, dysregulated cellular signaling pathways, and oxidative stress. These factors collectively promote OA progression and establish a vicious cycle, ultimately resulting in joint structural destruction. Current OA treatment typically involves using nonsteroidal anti-inflammatory drugs and COX-2 selective inhibitors to reduce inflammation by blocking prostaglandin E2 synthesis. These agents are associated with toxicity and heightened risks of gastrointestinal bleeding (Park et al., 2006) and cardiovascular events. Consequently, natural metabolites are gaining attention for their potential therapeutic benefits and enhanced safety.

### 3.1 The regulatory effects of allicin on osteoarthritis

Inflammation is pivotal in OA progression, marked by increased pro-inflammatory cytokines like interleukin-1β (IL-1β), interleukin-6 (IL-6), and tumor necrosis factor-α (TNF-α) (Park et al., 2006), (Ansari et al., 2020), (Kapoor et al., 2011), (Keller et al., 2023). These cytokines trigger the expression of matrix metalloproteinases (MMPs), inflammatory mediators, and reactive oxygen species (ROS), resulting in ECM degradation and joint dysfunction. Allicin treatment notably inhibits the PI3K/Akt/NF-κB signaling pathway activation induced by IL-1β in chondrocytes, a key mechanism in its action against OA (Qian et al., 2018). Furthermore, in a mouse model of surgically induced osteoarthritis, allicin treatment significantly reduced cartilage destruction, demonstrated by lower Osteoarthritis Research Society International scores (Qian et al., 2018). *In vitro* studies show that allicin inhibits IL-1β-induced overexpression of inflammatory mediators like nitric oxide (NO), prostaglandin E2, TNF-α, and IL-6, along with enzymes such as inducible nitric oxide synthase (iNOS) and COX-2, in a dose-dependent manner, thus reducing inflammation and ECM degradation (Qian et al., 2018). This effect is achieved by reducing NF-κB p65 nuclear translocation and IκBα degradation, thereby blocking downstream pro-inflammatory gene transcription. Chondrocytes in mature cartilage possess the capacity to proliferate and repair damaged cartilage tissue. *In vitro* experiments demonstrated that a 36-h allicin treatment significantly decreased the proportion of chondrocytes in the G0/G1 phase and increased those in the S-phase, accompanied by a notable upregulation of cyclin D1, CDK4, and CDK6 protein and mRNA expression. These findings indicate that allicin enhances chondrocyte proliferation and aids in cartilage tissue repair (Li et al., 2015). Furthermore, allicin reverses the IL-1β-induced overexpression of MMP-13 and restores the synthesis of type II collagen (Col II) and aggrecan, thereby maintaining the integrity of cartilage structure (Qian et al., 2018). Allicin not only possesses anti-inflammatory properties but also provides protection via antioxidant pathways. Oxidative stress, a key pathological mechanism in OA, is marked by an imbalance between elevated ROS production and reduced antioxidant capacity (Ansari et al., 2020). Nuclear factor erythroid 2-related factor 2 (Nrf2) is a crucial antioxidant transcription factor vital for cartilage integrity, with its dysfunction causing redox imbalance (Hecker et al., 2014). Under normal physiological conditions, Nrf2 is inactivated by binding to its negative regulator, Keap1. Research indicates that allicin mitigates H_2_O_2_-induced oxidative stress, decreases inflammatory factor expression, promotes cartilage matrix synthesis, and prevents chondrocyte hypertrophic differentiation through the activation of the Keap1/Nrf2 pathway, thus slowing OA progression (Yang J. et al., 2020).

### 3.2 The regulatory effects of SAMC on osteoarthritis

During OA progression, the majority of cartilage undergoes progressive attrition over time, accompanied by an imbalance in the MMPs/TIMP-1 ratio, which leads to exacerbated degradation of Col II, a major component of the ECM. SAMC, a key water-soluble garlic derivative, exhibits antioxidant, anti-inflammatory, and antitumor properties (Zhu et al., 2017), (Li et al., 2017). SAMC dose-dependently suppresses MMP-9 and MMP-13 expression while upregulating TIMP-1, thereby restoring MMPs/TIMP-1 balance and attenuating Col II degradation (Yang et al., 2017). The SAMC-induced elevation in Col II protein expression, along with the reduction in its proteolytic product C2C, helps preserve cartilage structure integrity by indicating less articular cartilage destruction. Furthermore, SAMC treatment significantly elevates cytoplasmic IκBα levels and reduces nuclear p65 content, suggesting that it may exert anti-inflammatory effects through suppression of NF-κB pathway activation and downstream inflammatory signal transduction (Yang et al., 2017). Recent studies suggest that SAMC may have therapeutic potential in OA by influencing Nrf2 via the NOX4/NF-κB pathway modulation (Yang G. et al., 2020). *In vitro* experiments showed that SAMC improves the viability and proliferation of chondrocytes diminished by IL-1β stimulation. However, under unstimulated basal conditions, SAMC did not exhibit a proliferative effect on chondrocytes (Yang et al., 2017). Similarly, in surgically induced rat OA models and in IL-1β-stimulated chondrocytes, NADPH oxidase 4 (NOX4) was activated while the expression of Nrf2 and its negative regulator Keap1 decreased. Both low-dose (2 mM) and high-dose (5 mM) SAMC reversed this trend and alleviated inflammation, with the protective effect being particularly pronounced in the SAMC (5 mM) group. However, SAMC (5 mM) did not further activate Nrf2, and the specific mechanism underlying this observation requires further elucidation. SAMC enhances the expression of Nrf2-dependent antioxidant enzymes, mitigating ROS-induced cartilage collagen degradation and thereby alleviating OA. In Nrf2-knockout chondrocytes, SAMC treatment not only failed to reverse oxidative stress but instead further activated NOX4 expression, leading to an increased MMPs/TIMP-1 ratio and exacerbated Col II degradation. Furthermore, the inhibitory effects of SAMC on COX-2 and iNOS expression were abolished upon Nrf2 deletion (Yang G. et al., 2020). These findings indicate that the protective effects of SAMC on OA cartilage are partially dependent on Nrf2, with the mechanism potentially involving targeted regulation of Nrf2 through the NOX4/NF-κB signaling pathway.

### 3.3 The regulatory effects of DAS and DADS on osteoarthritis

Under inflammatory stimulation, the expression of COX-2 is upregulated in chondrocytes and synovial cells of human articular tissues (Lee et al., 2009), therefore, COX-2 overexpression in articular tissues represents a significant pathological feature of inflammatory arthropathies. In rat OA models and *in vitro* cellular experiments, DAS has been shown to significantly inhibit COX-2 expression induced by IL-1β or monosodium urate crystals in chondrocytes and synoviocytes. Inhibiting the NF-κB signaling pathway may mediate this effect, educing synovial inflammation and cartilage degradation, indicating DAS’s potential therapeutic value in OA treatment (Lee et al., 2009). Results from relevant *in vitro* studies have shown that DADS dose-dependently inhibits IL-1-induced expression of MMPs, such as MMP-1 and MMP-13, demonstrating its therapeutic potential to alleviate inflammation associated with OA (Williams et al., 2010). This study also indicated that DAS, in a manner similar to DADS, dose-dependently suppresses the expression of the aforementioned MMPs (Williams et al., 2010).

**FIGURE 2:**
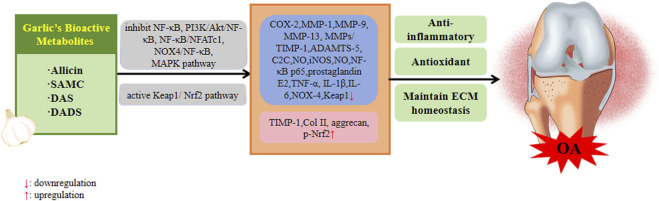
The impact of Allicin, SAMC,DAS and DADS on osteoarthritis regulation.

In summary, garlic and its bioactive metabolites have shown potential protective effects in preclinical models of OA through anti-inflammatory and antioxidant actions, ECM degradation inhibition, and tissue repair promotion (Figure 2). Notably, recent research has also revealed that garlic-derived exosome-like nanoparticles can attenuate IL-1β-induced ECM degradation, thereby protecting articular cartilage and thus alleviating the progression of OA *in vitro* and *in vivo*. This protective effect may result from inhibiting MAPK pathway activation during OA-related inflammation (Liu et al., 2025). Notably, almost all the findings cited in this section are from cell and animal studies, lacking sufficient human clinical evidence. Current data are still insufficient to confirm the clinical efficacy of garlic and its active metabolites in OA treatment. Thus, while preclinical studies suggest that garlic metabolites have potential protective effects, their translational potential for clinical application remains to be validated. Future high-quality human studies, such as well-designed randomized controlled trials, are needed to clarify their efficacy and safety.

## 4 The impact of garlic and its active metabolites on osteoporosis regulation

Osteoporosis (OP) is a systemic skeletal condition marked by diminished bone mass and deteriorating bone microarchitecture, leading to reduced bone strength and heightened fracture risk. Osteoporosis is classified into two primary categories: primary and secondary. Primary osteoporosis mainly affects the elderly and postmenopausal women (Rachner et al., 2011). OP generally begins around age 40, with a significantly higher incidence in women, particularly as postmenopausal osteoporosis, which is the most prevalent form. In addition to baseline factors like bone volume, strength, and density, the gender difference is mainly due to increased bone turnover and reduced bone density caused by the rapid drop in estrogen levels after menopause. The primary pathological mechanism of OP is the imbalance of bone homeostasis, characterized by disrupted equilibrium between osteoclast-driven bone resorption and osteoblast-driven bone formation. When bone resorption persistently exceeds bone formation, it results in progressive bone loss. Contemporary pathophysiological research has revealed that the process of bone loss involves the complex regulation of various cells, molecules, and signaling pathways. Multiple mechanisms, including hormonal imbalances, dysregulation of pro-inflammatory cytokines, genetic factors, and aging, collectively contribute to the progression of OP, ultimately leading to the destruction of bone microarchitecture, manifested as trabecular microfractures, increased porosity of cortical bone, and a consequent elevation in fracture risk.

### 4.1 The regulatory effect of garlic oil extract on osteoporosis

Postmenopausal osteoporosis is the most prevalent age-related bone loss, primarily resulting from ovarian failure and estrogen deficiency (Yao et al., 2025). Post-menopausal estrogen decline enhances bone resorption via several mechanisms: direct activation of NF-κB signaling in osteoclasts, reduced osteoprotegerin (OPG) expression, increased RANKL/RANK signaling, and diminished estrogen-mediated inhibition of osteoblast apoptosis (Yu et al., 2024). Garlic oil extract has been demonstrated to prevent weight gain and bone loss in ovariectomized mouse models, which simulate ovarian hormone deficiency (Mukherjee et al., 2004). Garlic oil extract effectively mitigates increased osteoclastic activity and bone resorption post-ovariectomy, lowers serum levels of high bone turnover markers like alkaline phosphatase and tartrate-resistant acid phosphatase, and reduces urinary excretion of calcium, phosphorus, and hydroxyproline, indicating a protective effect on bone health. Traditional treatment for postmenopausal osteoporosis involves estrogen replacement therapy or combined estrogen-progestogen therapy. Although this method alleviates menopause-related osteoporosis and its complications, long-term use may increase the risks of embolism or coronary event, stroke, and breast cancer (Bofill Rodriguez et al., 2025). Animal studies indicate that the cholesterol-lowering drugs lovastatin and simvastatin may also have bone-protective properties. Local subcutaneous injection of these two drugs promotes calvarial bone formation in mice, while oral administration increases cancellous bone mass in rats. *In vitro* experiments have demonstrated that lovastatin inhibits osteoclast formation and attenuates bone damage in mouse models (Mundy et al., 1999). However, statins do not exhibit osteogenic effects at conventional doses, and while high doses exert bone-protective and osteogenic effects, they may induce myotoxicity, hepatotoxicity, and adverse reactions such as gastrointestinal and sleep disturbances, limiting their long-term application (Abdul-Majeed et al., 2012). Preclinical evidence fromovariectomized rat models of osteoporosis suggests that garlic oil extract demonstrates clear anti-osteoporotic potential and benefits for maintaining skeletal health. In these models, garlic oil extract partially restores serum estrogen levels and promotes intestinal calcium transfer, thereby counteracting bone mineral loss caused by ovarian hormone deficiency, a mechanism potentially related to its phytoestrogenic effects (Mukherjee et al., 2006a), (Mukherjee et al., 2006b). Furthermore, garlic may inhibit the progression of bone tissue degeneration in ovariectomized rats by modulating immune cell function, particularly by affecting peritoneal macrophages and lymphocytes involved in the pathogenesis of hypogonadal osteoporosis (Mukherjee et al., 2007).

### 4.2 The regulatory effects of allicin, alliin, DADS, and DATS on osteoporosis

Oxidative stress significantly contributes to OP progression by accumulating ROS, which impairs osteoblast function and activates osteoclasts. Allicin enhances bone formation by reducing oxidative stress-induced osteoblast damage through activation of the PI3K/AKT and CREB/ERK signaling pathways, improving mitochondrial function, and inhibiting apoptosis, thereby enhancing bone formation capacity (Ding et al., 2016), (Liu et al., 2020). In contrast, alliin and DADS primarily exert bone-protective effects by inhibiting the aberrant differentiation and function of osteoclasts. Under homeostatic conditions, RANKL from osteoblasts and osteocytes binds to RANK on osteoclasts and their precursors, triggering their activation, fusion, and differentiation into mature multinucleated osteoclasts, thereby increasing bone resorption activity (Armas and Recker, 2012). Concurrently, OPG, produced by osteoblasts and osteogenic stromal stem cells, functions as a decoy receptor for RANKL. OPG competitively binds to RANKL, preventing its interaction with RANK, which inhibits osteoclast differentiation and activation, thus protecting the skeleton from excessive bone resorptionn (Boyce and Xing, 2007), (Yasuda, 2021). However, under inflammatory conditions, an elevated RANKL/OPG ratio and excessive osteoclast activation are observed. Alliin suppresses RANKL-induced osteoclastogenesis and bone resorption by downregulating Nox1 expression, leading to reduced ROS production and inhibition of the c-Fos/NFATc1 signaling pathway (Chen et al., 2016). DADS inhibits osteoclastogenesis by suppressing NF-κB and STAT3 pathways, reducing p65 subunit and STAT3 phosphorylation, and blocking NFATc1 activation and its interaction with NF-κB p65. Simultaneously, DADS decreases the production of pro-inflammatory factors like IL-1β, IL-6, TNF-α, and NO, thereby further inhibiting osteoclast formation (Yang et al., 2019). This dual action of DADS-directly inhibiting osteoclastogenesis and indirectly reducing inflammatory factors-significantly alleviates bone resorption in LPS-induced inflammatory models. DATS enhances mRNA and protein levels of osteogenic factors Runx2 and β-catenin, thereby activating the Wnt/β-catenin signaling pathway for osteogenesis, while concurrently reducing RANKL and TRAF6 expression to suppress osteoclast differentiation. Through these mechanisms, DATS improves trabecular bone microarchitecture, promotes collagen synthesis, and increases bone mineral density and bone volume fraction, thereby exerting anti-osteoporotic effects (Zhang et al., 2023).

Clinical studies further support the potential therapeutic value of garlic and its bioactive metabolites. A study found that garlic tablets with garlic powder and allicin significantly lowered serum levels of pro-inflammatory cytokines, such as TNF-α, IL-1β, and IL-6, in postmenopausal women with osteoporosis. This suggests a potential mechanism for reducing bone loss by inhibiting osteoclastogenesis and extending osteoclast lifespan (de Molon et al., 2018), (Mozaffari-Khosravi et al., 2012). Furthermore, garlic consumption has been shown to decrease levels of oxidative stress biomarkers in postmenopausal women. DAS and DADS, key contributors to garlic’s antioxidant properties, effectively suppress ROS production, restore SOD and GSH-Px activities in osteoblasts, and decrease apoptosis rates, thus improving osteoblast survival under oxidative stress conditions (Ahmadian et al., 2017). The study suggests that garlic and its active metabolites regulate bone metabolism by enhancing osteoblast activity and suppressing osteoclast formation, thus achieving anti-osteoporotic effects through balancing these cell functions.

**FIGURE 3:**
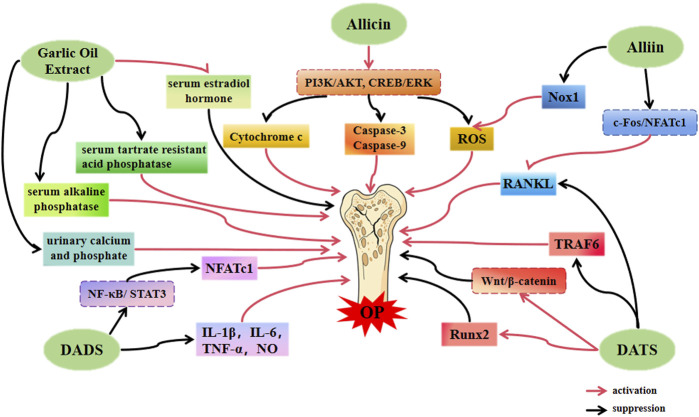
The impact of garlic oil extract, Allicin,Alliin, DADS and DATS on osteoporosis regulation.

In summary, garlic and its bioactive metabolites exert multifaceted regulatory effects in the context of osteoporosis by inhibiting osteoclast differentiation and function, enhancing osteoblast activity, and maintaining bone metabolic homeostasis. These effects are facilitated by antioxidant and anti-inflammatory mechanisms, alongside the regulation of critical signaling pathways such as NF-κB/STAT3-NFATc1, PI3K/AKT, and CREB/ERK. These findings provide a novel theoretical basis and potential therapeutic strategies for the treatment of OP (Ahmadian et al., 2017) (Figure 3).

## 5 The impact of garlic and its active metabolites on intervertebral disc degeneration regulation

Intervertebral disc degeneration (IVDD) is a progressive and irreversible condition marked by the deterioration of disc tissue, influenced by aging, mechanical stress, genetic factors, and metabolic changes. The intervertebral disc, serving as the shock-absorbing structure of the spine, is a fibrocartilaginous tissue connecting adjacent vertebrae. The intervertebral disc consists of three primary components: the peripheral annulus fibrosus (AF), the central gelatinous nucleus pulposus (NP), and the superior and inferior cartilaginous endplates. The AF is a ring-like structure enveloping the NP and is rich in collagen. The core NP has a gel-like consistency with a water content of 66%–86%, relying on its main ECM components—proteoglycans and type II collagen—to retain water and maintain its gel-like properties (Nedresky et al., 2023). IVDD is characterized by ECM degradation, reduced water and proteoglycan content, AF structural disruption, and diminished NP cell number and function (Yu et al., 2021). This process is often accompanied by enhanced oxidative stress and inflammation (Krut et al., 2021), consequently leading to a decline in the biomechanical properties of the intervertebral disc. Structural changes such as intervertebral space narrowing, osteophyte formation, annulus fibrosus fissures, and disc herniation can lead to clinical manifestations like low back pain and spinal stenosis (Xin et al., 2022).

The core pathological mechanism of IVDD is an imbalance between ECM synthesis and degradation within the NP. Oxidative stress and inflammation are key drivers in the progression of IVDD, triggered by factors like genetics, aging, and mechanical loading (Lu et al., 2023), (Chen et al., 2025), (Zhang et al., 2025), (Sao and Risbud, 2024). The intervertebral disc tissue primarily relies on anaerobic glycolysis for energy production (Wu et al., 2021), (Kim et al., 2021), a metabolic state that inherently predisposes it to increased ROS generation (Li et al., 2020). As age increases and IVDD progresses, the disc’s endogenous antioxidant defense system deteriorates, resulting in mitochondrial dysfunction and ROS accumulation (Chen et al., 2024). The excessive accumulation of ROS activates a series of MMPs and inhibits the expression of TIMPs, thereby accelerating ECM degradation. Direct evidence from IVDD-specific models indicates that allicin may protect against disc degeneration. Allicin has been shown to reduce oxidative stress and mitochondrial apoptosis in NP cells (Xi et al., 2020). Subsequent studies demonstrated that an ROS-responsive hydrogel containing allicin effectively prevents NP cell apoptosis and ECM degradation, while enhancing NP cell growth, proliferation, and repair. In animal models, this treatment significantly delayed IVDD and maintained disc morphology and matrix integrity (Zhang et al., 2025). In the study’s cellular experiments, H_2_O_2_ treatment significantly decreased Bcl-2 expression while increasing Bax, caspase-3, and caspase-9 levels, thus inducing apoptosis. Allicin treatment reversed the alterations in protein expression. The results indicate that allicin could slow IVDD progression by safeguarding NP cells against apoptosis caused by oxidative stress. In models of spinal cord injury, aged garlic extract has been demonstrated to reduce oxidative stress, inhibit the production of pro-inflammatory factors, alleviate mitochondrial dysfunction, and suppress caspase-3 activity in models of spinal cord injury, suggesting that it may possess similar therapeutic activity against intervertebral disc degeneration (Cemil et al., 2012)^,^ (Cemil et al., 2016). These findings suggest that aged garlic extract might possess similar therapeutic activity against intervertebral disc degeneration, but this remains to be directly tested in IVDD models.

Elevated ROS levels can activate inflammation-related signaling pathways, particularly NF-κB, leading to the release of pro-inflammatory factors (Lu et al., 2023), (Chen et al., 2025). Pro-inflammatory cytokines like TNF-α and IL-1β contribute to disc and cartilage matrix degradation by enhancing matrix-degrading enzymes, such as MMPs and ADAMTS (Binch et al., 2016), while also inhibiting aggrecan synthesis through the suppression of connective tissue growth factor expression (Tran et al., 2010), (Tran et al., 2014). Furthermore, pro-inflammatory cytokines such as TNF-α and IL-1β can directly compromise the survival and function of chondrocytes and NP cells in the intervertebral disc, while also enhancing oxidative stress, resulting in increased ROS accumulation. This creates a vicious cycle that significantly accelerates the destructive process within the disc (Purmessur et al., 2013), (Maidhof et al., 2014), (Ponnappan et al., 2011), (Gruber et al., 2014). Garlic and its primary bioactive metabolites exhibit antioxidant and anti-inflammatory properties. Their influence on signaling pathways, including PI3K/Akt/NF-κB, NOX4/NF-κB, and Nrf2/ARE, boosts cellular antioxidant defenses, suppresses NF-κB-mediated inflammatory signaling, and reduces pro-inflammatory cytokines such as TNF-α and IL-1β expression levels (Qian et al., 2018), (Yang G. et al., 2020), (Sun et al., 2021). Although these mechanisms have been characterized largely in osteoarthritis or chondrocyte models, they might hypothetically alleviate the negative impacts of the disc’s local inflammatory microenvironment. In OA studies, DADS dose-dependently inhibits the overexpression of MMP-1, MMP-3, and MMP-13 induced by inflammatory cytokines, thus reducing ECM catabolism in articular cartilage (Williams et al., 2010). This finding suggests that garlic extracts may also have potential protective effects against inflammation-related IVDD, but direct evidence in IVDD models is currently lacking.

**FIGURE 4:**
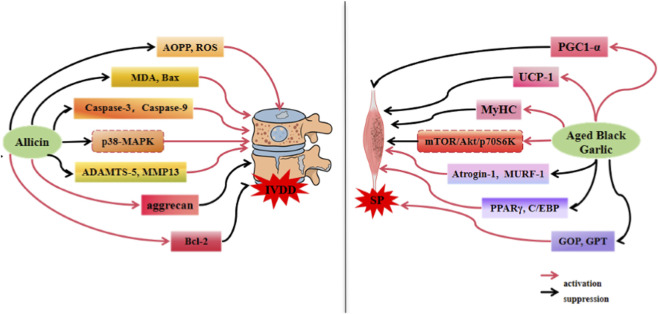
The impact of aged garlic extract, Allicin on intervertebral disc degeneration regulation and aged black garlic on sarcopenia regulation.

In summary, direct *in vitro* and *in vivo* evidence from IVDD-specific studies supports that allicin can delay IVDD progression by inhibiting NP cell apoptosis and reducing oxidative damage (Figure 4). However, there is currently no direct evidence supporting the therapeutic effects of garlic’s other bioactive metabolites on inflammation or ECM degradation specifically in IVDD. The shared antioxidant and anti-inflammatory properties observed in osteoarthritis and spinal cord injury models require direct validation in disease-specific IVDD models before any conclusive claims can be made.

## 6 The impact of garlic and its active metabolites on sarcopenia regulation

Sarcopenia (SP) is an age-related geriatric syndrome characterized by the gradual decline in skeletal muscle mass, muscle strength, and physical function. It significantly increases the risks of frailty, falls, disability, and mortality, imposing a substantial disease burden (Kirk et al., 2024), (Geriatrics Branch of the Chinese Medical Association, 2025). Epidemiological research shows that SP affects about 10%–27% of individuals worldwide aged 60 and above. The global population’s rapid aging is expected to increase the number of individuals with SP to 500 million by 2050 (Petermann-Rocha et al., 2022). Thus, identifying effective strategies for preventing and treating SP is urgently required. The pathogenesis of SP involves the interplay of multiple factors, including oxidative stress, chronic inflammation, insulin resistance, imbalance in protein metabolism, and mitochondrial dysfunction (Cho et al., 2022), (Wang et al., 2025). Recent perspectives indicate that gut microbiota dysbiosis may play a role in the onset and progression of SP. This is suggested to result from decreased production of anabolic substrates like short-chain fatty acids, antioxidant vitamins, and amino acids, leading to reduced anti-inflammatory and antioxidant capacity, limited nutritional signals to skeletal muscle, and increased protein breakdown with inhibited synthesis (Ticinesi et al., 2020).

Chronic inflammation, marked by increased pro-inflammatory cytokines, is a primary contributor to cellular degradation. Elevated pro-inflammatory cytokines, including IL-1, IL-6, TNF-α, and CRP, contribute to mitochondrial dysfunction in skeletal muscle, increasing ROS production and oxidative stress, which subsequently accelerates telomere erosion and cellular senescence (Petermann-Rocha et al., 2022). Conversely, pro-inflammatory factors may excessively activate the ubiquitin-proteasome cascade by triggering signaling pathways like NF-κB and p38 MAPK, leading to enhanced muscle proteolysis (Nishikawa et al., 2021), (Picca and Calvani, 2021). Concurrently, IL-6 can simultaneously cause insulin resistance, suppress the Akt/mTOR signaling pathway, and hinder muscle protein synthesis (Nishikawa et al., 2021), (Bilski et al., 2022). Research indicates that garlic and its bioactive metabolites mitigate oxidative stress by neutralizing reactive oxygen species, preventing lipid peroxidation, and boosting the body’s antioxidant defenses (Ahmadian et al., 2017), (Shang et al., 2019). Additionally, they exhibit anti-inflammatory properties by inhibiting the production of inflammatory mediators like NO, TNF-α, and IL-1 (Yang et al., 2019), (Rahman, 2003). In addition, evidence suggests that garlic extract can attenuate skeletal muscle atrophy by modulating the proteolytic system in mice and has shown potential in inducing skeletal muscle cell proliferation (Shang et al., 2019), (Gupta et al., 2020). These experimental results have shown potential protective effects of garlic’s active metabolites on muscle health, but these effects have not yet been confirmed in SP models.

Epidemiological studies indicate a significant inverse relationship between raw garlic and onion consumption and the prevalence and incidence of SP among the elderly population (Wang et al., 2025). Clinical trials have demonstrated the beneficial effects of garlic; a randomized controlled trial revealed that 12 weeks of garlic powder supplementation significantly increased skeletal muscle mass in participants. A single-blind crossover study found that 4 weeks of garlic supplementation significantly improved systemic antioxidant capacity and decreased markers of exercise-induced muscle damage (Sangouni et al., 2020). Animal and *in vitro* experiments have elucidated some of the underlying molecular mechanisms (Shang et al., 2019), (Lee et al., 2019). Additionally, a study confirmed that aged black garlic and aged black elephant garlic activate the Akt/mTOR/p70S6K signaling pathway via their key active metabolites, S-methyl-L-cysteine and L-proline. This pathway’s activation suppresses muscle atrophy protein expression, enhances muscle synthesis and differentiation, and boosts mitochondrial function, thereby mitigating obesity-related muscle atrophy induced by a high-fat diet in mice (Chae et al., 2023). Although the muscle atrophy induced by these diseases is biologically related to age-related SP, it is not interchangeable with it. Thus, these results are mechanistically informative but not directly translatable. 1n a cancer cachexia model, the garlic-derived sulfur metabolite Z-ajoene attenuated skeletal muscle atrophy and suppressed the key atrogenes MAFbx and MuRF1 and the inflammatory myokines such as IL-6 and myostatin (Damluji et al., 2023). However, cancer cachexia is driven by tumor-derived systemic inflammation, and its etiology is fundamentally different from SP; therefore, these experimental results can only serve as indirect mechanistic references and cannot be directly extrapolated to SP models.

In summary, evidence spanning epidemiological, clinical, and preclinical studies indicates that garlic and its bioactive constituents may protect muscle health through multiple mechanisms (Figure 4). Nevertheless, these findings have not directly established therapeutic efficacy against SP, and future disease-specific investigations are required for validation.

## 7 Conclusion and prospect

This article reviews the regulatory effects and mechanisms of garlic and its primary bioactive metabolites in OA, OP, IVDD, and SP. Garlic’s bioactive metabolites delay disease progression by exerting anti-inflammatory, antioxidant, and cell signaling regulatory effects, and enhancing bone metabolic balance through multi-target and multi-pathway mechanisms. Allicin alleviates chondrocyte inflammation and ECM degradation in OA by inhibiting the PI3K/Akt/NF-κB signaling pathway. SAMC may influence Nrf2 through the NOX4/NF-κB pathway, providing antioxidant and anti-inflammatory benefits. In osteoporosis, garlic metabolites disrupt the RANKL/RANK/OPG system to inhibit osteoclast differentiation and enhance osteogenesis via pathways like Wnt/β-catenin, thus preserving bone metabolic balance. In IVDD, garlic extract and its metabolites have demonstrated potential in protecting NP cells, reducing apoptosis, and delaying degeneration through anti-inflammatory, antioxidant, and MMP-inhibitory activities. In SP, observational studies suggest that garlic consumption is associated with lower prevalence and incidence; however, current evidence from interventional studies or preclinical experiments remains limited, and further research is needed for validation. Overall, currentl evidence suggests that garlic and its bioactive metabolites possess potential protective effects against degenerative musculoskeletal diseases. However, the available evidence is primarily derived from *in vitro* and animal studies, and there remains a lack of high-quality, disease-specific randomized controlled trials to confirm their clinical efficacy and safety. Although garlic has a historical application in the prevention and treatment of various diseases as a natural product, systematic research targeting degenerative musculoskeletal diseases is still insufficient, and the elucidation of underlying mechanisms as well as clinical translational evidence remain incomplete. Future efforts urgently require in-depth mechanistic validation and well-designed large-scale clinical studies to refine the theoretical framework and establish a solid evidence-based medicine foundation.

An emerging perspective suggests that sulfur-containing metabolites in garlic may exert core protective effects in degenerative musculoskeletal diseases through the release of the endogenous gasotransmitter hydrogen sulfide (H_2_S) (Rodrigues and Percival, 2019). H_2_S itself has been demonstrated to possess protective properties against various degenerative musculoskeletal disorders (Behera et al., 2019), (Song et al., 2024). This mechanism connects a traditional medicinal plant with modern gasotransmitter biology, offering a novel avenue for elucidating its pharmacodynamic material basis. Furthermore, garlic has long been incorporated into traditional Chinese medicine formulas. For instance, it is a component of Qing’e Wan, a classical formula comprising *Psoralea corylifolia*, *Eucommia ulmoides*, walnut kernel, and garlic, which is traditionally indicated for kidney deficiency manifesting as lumbar pain and bone weakness with reduced physical strength. This formula is now also commonly used as an adjunctive therapy for postmenopausal osteoporosis, reflecting the traditional value of garlic in musculoskeletal health (Xiong et al., 2022). Traditional Chinese medicine formulas, known for their multi-component and multi-target holistic regulation, offer distinct benefits in alleviating clinical symptoms of degenerative musculoskeletal diseases. Future studies should investigate the synergistic effects and regulatory mechanisms of garlic in these formulas, offering new insights for the integrated prevention and treatment of degenerative musculoskeletal diseases through traditional and modern medical approaches.
